# Cerebral microemboli in mini-sternotomy compared to mini- thoracotomy for aortic valve replacement: a cross sectional cohort study

**DOI:** 10.1186/s13019-021-01509-8

**Published:** 2021-05-24

**Authors:** Marija Bozhinovska, Matej Jenko, Gordana Taleska Stupica, Tomislav Klokočovnik, Juš Kšela, Matija Jelenc, Matej Podbregar, Andrej Fabjan, Maja Šoštarič

**Affiliations:** 1 Clinical Department of Anaesthesiology and Perioperative Intensive Therapy, University Medical Centre Ljubljana, Zaloska 2, 1000 Ljubljana, Slovenia; 2 University of Ljubljana, Faculty of Medicine, Ljubljana, Slovenia; 3 Clinical Department of Cardiovascular Surgery, University Medical Centre Ljubljana, Ljubljana, Slovenia; 4 Department of Internal Intensive Medicine, General and Teaching Hospital, Celje, Slovenia; 5 Clinical Department of Neurology, University Medical Centre Ljubljana, Ljubljana, Slovenia

**Keywords:** Aortic valve replacement, Minimal invasive surgery, Mini-sternotomy, Mini-thoracotomy, Transcranial Doppler, Microembolic signals, Cognitive test

## Abstract

**Background:**

Recently adopted mini-thoracotomy approach for surgical aortic valve replacement has shown benefits such as reduced pain and shorter recovery, compared to more conventional mini-sternotomy access. However, whether limited exposure of the heart and ascending aorta resulting from an incision in the second intercostal space may lead to increased intraoperative cerebral embolization and more prominent postoperative neurologic decline, remains inconclusive. The aim of our study was to assess potential neurological complications after two different minimal invasive surgical techniques for aortic valve replacement by measuring cerebral microembolic signal during surgery and by follow-up cognitive evaluation.

**Methods:**

Trans-cranial Doppler was used for microembolic signal detection during aortic valve replacement performed via mini-sternotomy and mini-thoracotomy. Patients were evaluated using Addenbrooke’s Cognitive Examination Revised Test before and 30 days after surgical procedure.

**Results:**

A total of 60 patients were recruited in the study. In 52 patients, transcranial Doppler was feasible. Of those, 25 underwent mini-sternotomy and 27 had mini-thoracotomy. There were no differences between groups with respect to sex, NYHA class distribution, Euroscore II or aortic valve area. Patients in mini-sternotomy group were younger (60.8 ± 14.4 vs.72 ± 5.84, *p* = 0.003), heavier (85.2 ± 12.4 vs.72.5 ± 12.9, *p* = 0.002) and had higher body surface area (1.98 ± 0.167 vs. 1.83 ± 0.178, *p* = 0.006). Surgery duration was longer in mini-sternotomy group compared to mini-thoracotomy (158 ± 24 vs. 134 ± 30 min, *p* < 0.001, respectively). There were no differences between groups in microembolic load, length of ICU or total hospital stay. Total microembolic signals count was correlated with cardiopulmonary bypass duration (5.64, 95%CI 0.677–10.60, *p* = 0.027). Addenbrooke’s Cognitive Examination Revised Test score decreased equivalently in both groups (*p* = 0.630) (MS: 85.2 ± 9.6 vs. 82.9 ± 11.4, *p* = 0.012; MT: 85.2 ± 9.6 vs. 81.3 ± 8.8, *p* = 0.001).

**Conclusion:**

There is no difference in microembolic load between the groups. Total intraoperative microembolic signals count was associated with cardiopulmonary bypass duration. Age, but not micorembolic signals load, was associated with postoperative neurologic decline.

**Trial registry number:**

clinicaltrials.gov, NCT02697786 14.

**Supplementary Information:**

The online version contains supplementary material available at 10.1186/s13019-021-01509-8.

## Introduction

Degenerative aortic valve stenosis is the most common valvular cardiac disease in developed countries and affects more than 4% of North American and European citizens [1, 2]. Surgical aortic valve replacement (AVR) with the use of cardiopulmonary bypass (CPB) is one of several treatment options. Despite improvements in surgical techniques and postoperative medical care, neurological impairment remains a major complication after cardiac surgery [3]. Post-cardiac surgery brain injuries are categorized in two groups: The first group includes patients with visible computed tomography (CT) findings and neurological decline such as stroke, while the second group includes patients with delirium and neurocognitive decline [4, 5]. Mechanisms of brain injury after cardiac surgery are related to impaired cerebral blood flow, hyperthermia, atrial fibrillation, genetic predisposition, and systemic inflammatory response (SIRS) associated with CPB [5]. In addition, intraoperative cerebral embolization appears to be an important mechanism of such injury, as cerebral emboli, both gaseous or solid, that can cause ischemia, inflammation, and edema, consequently causing cerebral micro infarctions [5]. Neurological complications may arise despite successful cardiac surgical procedure and can significantly influence a patient’s general condition and postoperative quality life [6–9].

During the last decade, a conventional full sternotomy approach for surgical AVR has been widely replaced by a less aggressive mini-sternotomy (MS) access due to multiple benefits, such as reduced postoperative morbidity, mortality, and pain, while providing faster recovery, a shorter hospital stay, and better cosmetic results [10, 11]. Furthermore, in recent years, several expert surgical teams have reported on successful AVR procedures performed through technically more challenging mini-thoracotomy (MT) approach.

However, while such surgical approach is less invasive with better cosmetics results than MS, concerns have been raised by a number of clinicians that a limited exposure of the aorta and the heart achieved by an incision through a second intercostal space may potentially lead to incomplete heart and aorta de-airing at the end of the procedure, increasing the risk for cerebral microembolization [12].

Since postoperative neurological complications due to microembolization can significantly influence patient’s general condition and postoperative quality life, the objectivization of the incidence of microembolization after MT AVR would be of outmost importance in order for the clinicians to offer their patients the best suited surgical AVR with an optimal ratio between minimally invasiveness and perioperative complications. Thus, the aim of our study was to compare intraoperative MES between well accepted MS approach and a novel, less aggressive MT access for surgical AVR and to test the relationship between intraoperative microembolic signals (MES) and postoperative cognitive decline in both groups of patients.

## Methods

This was a prospective, observational; single-center clinical trial was carried out at the Clinical Department of Anesthesiology and Perioperative Intensive Therapy, Division of Cardiovascular Anaesthesia and Intensive therapy, and Clinical Department of Cardiovascular Surgery at the University Medical Centre in Ljubljana (Slovenia), between March, 2016 and December, 2018. Approval for the trial was obtained from the National Medical Ethics Committee (Affiliation: Ministry of Health of Republic of Slovenia; approval number: 22 k/04/15) and a written informed consent was obtained from each patient before enrolment into the study. The trial was conducted in accordance with the Helsinki Declaration and registered at ClinicalTrials.gov (NCT02697786) before patient recruitment was started.

*Power of study* calculation was based on the assumption that the absolute number of MES would be around 100/procedure (SD ± 25 MES) [12] and any change in the mean difference of 20 MES/procedure or greater would suffice as a clinically relevant effect for a two-sided test. To achieve 80% statistical power with a significance level (*α*) of 5%, the ClinCalc sample size calculator (*clincalc.com/stats/samplesize.aspx*) defined our need as 25 patients per group, indicating a total of 50 patients across the two treatment groups. Considering an estimated 10% drop-out rate due to a poor trans-temporal window for transcranial Doppler (TCD), 60 patients were recruited to avoid risk of low power.

### Inclusion criteria

Patients> 18-years-old, ​II or III class according American Society of Anesthesiology (ASA) classification, diagnosed with isolated severe high-gradient aortic stenosis according to ESC/EACTS 2017 guidelines (AVA less than 1.0 cm^2^, mean gradient>_40 mmHg and indexed aortic valve area less than 0,6 cm^2^/m), who were referred to Clinical Department of Cardiovascular Surgery at the University Medical Centre in Ljubljana for mini-AVR, were eligible for recruitment.

### Exclusion criteria

Patients with a poor acoustic window, epilepsy, psychiatric illness, carotid pathology, pathology of coronary arteries, brain stroke and alcohol abuse were excluded from the study.

### Anesthesiological procedures

All patients in the OR received standard and extended hemodynamic monitoring, including electrocardiogram, arterial cannula for invasive hemodynamic measurements (FloTrac™ System, Edwards Lifesciences; USA), and central venous catheter (PreSep Central Venous Oximetry catheter; Edwards Lifesciences, USA) in the jugular vein to monitor cardiac index, systemic vascular resistance index, and central venous pressure. Intraoperative neuromonitoring included near infrared spectroscopy (NIRS) (INVOS Cerebral/Somatic Oximeter, Medtronic, USA) to follow cerebral oxygenation and bispectral index (BIS VISTA™ Brain Monitoring System, Medtronic, USA) to follow the depth of anaesthesia. Patients were intubated with fentanyl 5–10 μg/kg, propofol 1–2 mg/kg, and rocuronium bromide 0.6 mg/kg, and were mechanically ventilated with a tidal volume 6–8 mL/kg ideal body weight. Patients were operated on under total intravenous anaesthesia with propofol, according to bispectral index values, for the depth of anaesthesia, and remifentanil (0.3–0.6 μg/kg/min). After sedation, TCD probes were placed. After setting the baseline values on hemodynamic and cerebral monitoring tools, patient’s hemodynamic parameters were recorded continuously during surgery and acid base management was performed according to the pH stat regimen. An aortic valve was replaced during CPB with non-pulsatile flow of 2.2 to 2.4 L/m^2^ BSA in mild hypothermia. During weaning from CPB, patients were supported with vasopressors or inotropes aiming for a mean arterial pressure (MAP) of 70–75 mmHg in the perioperative and later throughout the postoperative course. In clinically stable condition, patients were transferred to the cardiovascular ICU, sedated and intubated on mechanical ventilation. Postoperative pain was treated with a catheter into the wound, filled with ropivacaine, as well as intravenous paracetamol and metamizole. Weaning from mechanical ventilation was performed when extubating criteria were fulfilled, meaning: an awake and cooperative patient, with end tidal CO_2_of 4–6 kPa, haemoglobin oxygen saturation > 96% and hemodynamically stabile with normal core temperature. When patients were without vasopressors/ inotropes, in good general condition, and without signs of delirium or infections, they were transferred to a step-down unit and sequentially discharged from the hospital. Patient follow-up after discharge from the hospital was conducted by telephone 30 days after surgery, with the focus on late postoperative morbidity and mortality.

### Trans-cranial doppler

Bilateral monitoring of the middle cerebral arteries was performed using multi-range, multifrequency 2 MHz TCD probes (LookiWaki, Atys Medical, France). Signals were analyzed on-line and recoded. The back scattered embolic high intensity transient signals (HITS) were counted [13–15]. HITS were classified into MES and artifacts using the criteria recommended in the International Consensus Group on Microembolus Detection [13]. Software EADS 1.32 (Atys Medical, France) was used for off-line re-analysis. The maximal peak systolic velocity (V_max_) through the middle cerebral artery during surgery was also recorded.

### Surgical mini-invasive AVR techniques

All minimally invasive surgical procedures were performed by three experienced cardiac surgeons (K.T., J.M., K. J.). Patients were divided into two groups, MS and MT groups. In the MS group, classical J-shaped upper mini-sternotomy into the third intercostal space was performed [16]. In the MT group, anterior right mini-thoracotomy was performed through the second intercostal space. The Ljubljana MT approach was in details presented elsewhere [16–18]. Before aortic cannulation and cross clamping of the aorta an extensive TEE scan of theascending aorta was performed in every patient to exclude the presence of possible atherosclerotic plaques. In both groups, the ascending aorta was cannulated as cranially as possible and an EOPA arterial cannula (Medtronic, Inc., Minneapolis, MN, USA) was inserted. Central venous cannulation was accomplished by cannulating the superior vena cava using 22 Fr venous cannulas (Medtronic, Inc., Minneapolis, MN, USA) with one right angle cannula placed in the direction toward the head and a straight cannula placed in the right atrium [18]. Alternatively, a single 29 Fr flexible venous cannula (Optiflow, LivaNova PLC, London, UK) placed through the superior vena cava into the right atrium was used. CO_2_ was insufflated into the cardiothoracic wound through a gas diffuser (Linde, Slovenia) that provides an almost 100% CO_2_ atmosphere. CO_2_ was insufflated with flow set on 2 L/min. Selection of prosthetic valves was made according to patient age: in adults > 65 years we used biological valves and for younger than 65 years mechanical valves [19].

### Laboratory IL_6 analysis

For all laboratory analyses, blood samples were collected without additive. Serum was separated from clotted blood by centrifugation (1500×*g* for 10 min) and aliquots were stored at − 20 °C until analysis. Serum IL-6 at base line and 6 h after surgery were measured by chemiluminescent immunometric assays using Immulite automated analyser (Siemens Healthcare, Erlangen, Germany).

### Cognitive assessment

Addenbrooke’s Cognitive Examination Revised Test (ACE-R) was performed in each patient before surgery and up to 30 days after. ACE-R is a diagnostic and screening instrument comprised of 5 domains: attention and orientation, memory, verbal fluency, language, and visuo-spatial abilities [20, 21].

### Data collection

Preoperative data was collected (i.e. age, sex, body weight, body height, body surface area, NYHA class, left ventricular ejection fraction, aortic valve surface area, and renal function) from medical documentation. Surgical risk assessment was performed on the basis of EuroSCOREII (European System for cardiac Operative Risk Evaluation, http://www.euroscore.org/calc.htmL). Furthermore, intraoperative data (procedural time, CPB time, blood loss, inotropic/vasoactive drugs, acid-base status, data from the neuromonitoring tools) and postoperative period (length of ICU stay, intubation time, neurocognitive status, length of in-hospital stay), along with postoperative complications like bleeding, infections, hemodynamic instability, worsening of brain, kidney, respiratory, and liver function, including 30-day mortality were documented.

### Measurement time frame

To be able to follow the patient more precisely, we divided perioperative period into six parts: ***1st period***: from skin incision until aortic cannulation, ***2***^***nd***^***period***: from aortic cannulation until start of CPB, ***3rd period:*** the beginning of CPB until aorta clamping, ***4***^***th***^***period:*** from beginning of aorta clamping until aorta clamp removed, ***5***^***th***^***period:*** from aorta clamp removal until end of CPB, ***6th period:*** end of CPB until end of surgery.

### Outcome measures

*Primary outcome measure* was the MES count during MS and MT.

*Secondary outcome measures* were changes in cognitive score, hemodynamic variables (i.e., cardiac index) and IL − 6 levels.

*Other pre-specified outcome measures* included duration of postoperative mechanical ventilation, length of ICU stay, use of inotropic/vasoactive drugs, length of in-hospital stay, and 30-day mortality.

### Statistical analysis

Demographic and clinical baseline data were summarized according to mean and standard deviation for metric variables, or to absolute value and frequency for categorical variables. Mann-Whitney U and Chi-square tests were used, respectively.

Mixed model and repeated measures ANOVA were used to compare MES load between different time points and groups. Based on theoretical expectations, we created multivariable linear regression models for selected dependent variables: level of IL-6 tested 6 h after surgery, maximal deviation of NIRS oxygenation value from the baseline during surgical procedure, maximal median cerebral artery blood flow velocity during procedure, length of ICU stay (days), total duration of hospital stay (days), results of mental state examination after surgery, and the total intraoperative MES count. Binomial logistic regression was used to test the correlation of variables with postoperative incidence of delirium. The natural logof post-surgery IL-6 levels was used in all models to follow normality distribution, while IL-6 baseline levels were positive or negative. Because the nature of the study was the search for possible correlated indicators on selected dependent variables rather than hypothesis-driven, the *p*-value correction for multiple testing was not performed.

The software package: A Language and Environment for Statistical Computing (R core team, R foundation, Vienna, Austria, 2019) for statistical computation was used. *P*-values under 0.05 were considered statistically significant.

## Results

In total, 60 patients were recruited for the study, but eight patients were excluded due to poor trans-temporal window for TCD. We were left with 52 patients who underwent elective AVR, 25 assigned to the MS group and 27 to the MT group. Patient demographic characteristic and aortic valve pathology are presented in Table 1. There was no sex difference between MS and MT groups (71% male, 29% female, *p* = 0.458). There was also no difference in NYHA class distribution between the MS and MT groups (NYHA I.: 1 vs. 0, NYHA II.: 20 vs 20,NYHA III.: 4 vs 7, chi-square, *p* = 0.418, respectively). Compared to the MT group, patients in the MS group were younger (60.8 ± 14.4 years vs.72 ± 5.84 years, *p* = 0.003), heavier (85.2 ± 12.4 kg vs.72.5 ± 12.9 kg, *p* = 0.002), (Table 1). Euroscore II and aortic valve area were similar between groups. Patients in the MT group were more likely to be hypertensive compared to the MS group (*p* = 0.013).

**Table 1:** General data, echocardiography, anesthesia

	All (***n*** = 52)	MS (***n*** = 25)	MT (***n*** = 27)	***p***-value*
Age	66.6+/−12.1	60.8+/− 14.4	72+/−5.84	0.003
Gender				0.462
Male	37 (71)	19 (76)	18 (67)	
Female	15 (29)	6 (24)	9 (26)	
Height (cm)	170 ± 8.10	172 ± 8.3	168 ± 7.54	0.057
Weight (kg)	78.6 ± 14.1	85.2 ± 12.4	72.5 ± 12.9	0.002
BSA (m^2^)	1.90+/− 0.187	1.98+/− 0.167	1.83+/− 0.178	0.006
Hypertension,n (%)	41 (79)	16 (64)	25 (92,5)	0.013
Diabetes, n (%)	11 (21,1)	4 (16)	7 (25,9)	0.393
Creatinin (mg/dL)				
baseline	84.1+/− 23.6	83.7+/−14.7	84.6+/−21	0.533
GFR, ml/min	76.5+/− 14.4	80+/− 14.7	73.2+/−13.6	0.036
Euroscore	1.19+/−0.742	1.12+/− 0.734	1.26+/− 0.757	0.350
**Echocardiography**				
AVA (cm^2^)	0.772+/−0.138	0.8+/−0.131	0.74+/− 0.139	0.083
Mean gradient on	49+/−12.5	49.3+/− 12.5	48.6+/− 12.7	0.783
Aortic valve (mmHg)				
AVA index (cm^2^/m^2^)	0.408+/−0.0763	0.415+/− 0.0693	0.393+/− 0.0836	0.595
Ejection fraction (%)	63.3+/−10.8	61.8+/−8.83	64.7+/− 12.4	0.271
Velocity time integral	21.7+/−3.37	21.9+/−3.38	21.5+/−3.41	0.854
VTI in LVOT (cm)				
**Anesthesia**				
MAP mmHg-baseline	99.5+/−17.1	104+/− 14	95.4+/− 18.8	0.124
Fentanyl (mg)	0.522+/−0.227	0.548+/− 0.265	0.498+/− 0.187	0.777
Propofol (mg)	115+/−45.2	132+/−50.58	98.889+/−33.205	0.012
Rocuronium bromide (mg)	96.1+/−31.4	107.2+/− 37.807	85.741+/−19.54	0.028
Remifentanyl (mg)	4.88+/−1.67	5.468+/−1.457	4.338+/− 1.691	0.014
Propofol (gr)	1.36+/−0.521	1.487+/− 0.36	1.247+/− 0.620	0.004

MT-Mini thoracotomy, MS-Mini sternotomy, ACE-R Addenbrooke’s Cognitive Examination Test, AVA aortic valve area, VTI- velocity time integral, LVOT left ventricular outflow tract, MAP mean arterial pressure.

In the MS group, 11 mechanical valves and 14 bioprosthetic valves were implanted, while in the MT group, all implanted valves were bioprosthetic (Table 2).

**Table 2:** Perioperative characteristics and postoperative outcome

	All (***n*** = 52)	MS (***n*** = 25)	MT (***n*** = 27)	***p***-value*
Valve Type				<.001
Bioprosthetic valve, n(%)		14 (56)	27 (100)	
Mechanical valve, n (%)		11 (44)	0	
Surgery duration (min)	146 ± 30.2	158 ± 24.9	134 ± 30.5	<.001
CPB duration (min)	72.7 ± 23.3	81.1 ± 21.6	64.9 ± 22.4	<.001
Aortic X-clamp (min)	49.6 ± 17.4	60.8 ± 15.3	39.1 ± 12	<.001
Intubation time (hours)	7.44 ± 6.15	9.32 ± 7.65	5.7 ± 3.75	0.034
ICU stay (day)	1.94 ± 1.19	2.24 ± 1.48	1.67 ± 0.78	0.164
SUB ICU stay (day)	1.73 ± 0.992	1.76 ± 1.09	1.7 ± 0.912	0.92
Total hospital stay	9.44 ± 3.16	9.60 ± 2.97	9.30 ± 3.38	0.604
Delirium, n (%)	8 (15.3)	4 (16)	4 (14.8)	0.907
Pericardial effusion	5 (9.61)	4 (14.8)	1 (4)	0.138
Infections, n (%)	2 (3.83)	1 (4)	1 (3.70)	0.602
30-day Mortality, n(%)	0	0	0	

MT-Mini thoracotomy, MS-Mini sternotomy, CPB-cardiopulmonary bypass.

There was no statistically significant difference in MES count between the MS and MT groups (repeated measures ANOVA,*p* = 0.91167) (Additional Table S1).

In the 4th period, from aorta clamp-on until clamp removal, the highest MES load was detected in both groups compared to other time points (repeated measures ANOVA, *p* = 0.00288). The total intraoperative MES count was associated with CPB duration (Additional Table S2).

The cardiac index was lower in the MT compared to MS group at different periods of surgery: during the 1st period (from skin incision until aortic cannulation) (2.100 ± L/min/m^2^ vs.2.448 ± 0.712 L/min/m^2^, *p* = 0.045, respectively), the 2nd period (from aortic cannulation until start of CPB) (1.958 ± 0.509 L/min/m^2^ vs. 2.403 ± 0.872 L/min/m^2^, *p* = 0.012, respectively), the 3rd period (the beginning of CPB until aorta clamping (1.772 ± 0.479 L/min/m^2^ vs. 2.272 ± 0.814 L/min/m^2^, *p* = 0.016, respectively) and during the 6th period (end of CPB until surgery completion (2.228 ± 0.429 L/min/m^2^ vs. 2.665 ± 0.632 L/min/m^2^, *p* = 0.007, respectively).

There were no statistically significant differences in postoperative complications between the groups (Table 2). Overall, the MT group had a statistically significant shorter intubation time (*p* = 0.034) compared to the MS group, but no differences in lengths of ICU or in-hospital stays.

Compared to preoperative values, the total ACE-R score decreased postoperatively from 85.13 ± 5.41 to 82.11 ± 5.987 points (*p* < 0.05). There was no difference in change of ACE-R score between groups (*p* = 0.630); total ACE-R score decreased equivalently in the MS (85.2 ± 9.6 vs.82.9 ± 11.4 points, *p* = 0.012) and MT group (85.2 ± 9.6 vs. 81.3 ± 8.8 points, *p* = 0.001).

Multiple linear regressions, including surgery duration (min), CPB duration (min), cell saver amount (L), CRP 6 h after surgery, and total number of MES during surgery, revealed that MES count was an independent predictor of IL-6 level 6 h after surgery (Additional Table S3).

Average maximal velocity in the middle cerebral artery (OR 1.38 (1.03–1.83), *p* = 0.03) measured by TCD during surgery was an independent predictor of delirium development as measured by multiple linear regression including surgery duration, ACE-R after surgery, intraoperative NIRS deviation, and inclusion in MS/MT group (Additional Table S4).

Surgery and CPB duration each independently predicted ICU stay (Additional Table S5).

Age, but not MES load, was only independently associated with postoperative cognitive function testing (Additional Table S6).

## Discussion

The main finding of our study is that MT approach for surgical AVR is comparable to MS access in regards to MES load, indicating that MT represents a safe and efficient minimally invasive surgical approach for AVR, enabling a feasible surgical valve procedure through the second intercostal space without any division of the sternum. In both groups of patients, the highest MES load was detected in the surgical period from aorta clamp-on to aorta clamp removal. Total intraoperative MES count was associated with CPB duration. Abu Omar et al. showed that cerebral embolization is substantially reduced in patients operated off pump compared to on pump group [22]. Additionally, another study showed decreased number of microemboli in patients operated off pump for GABG, but they were not able to confirm the correlation between CPB or cerebral microremboli with cognitive decline [23].

The maximal peak systolic velocity in the middle cerebral artery during surgery was independently associated with development of postoperative delirium. We found no correlation between postoperative neurological decline and MES load. The only independent variable associated with postoperative neurological decline was patient age.

The minimally invasive AVR has evolved into an efficient treatment option, especially in experienced centers, providing greater patient satisfaction and lower complication rates [24]. Several studies from an experienced surgical center have shown that minimally invasive AVR approaches are safe and effective alternatives to conventional sternotomy [25, 26].

However, some authors have suggested that smaller incisions led to poor surgical filed exposure, potentially making the surgery technically more demanding and with longer operative times [27].

In our study, surgery duration was shorter in the MT group. This was in concordance with the findings of Olds et al. [25], who showed in a larger sample that the MT approach decreased operative time, length of hospital stay, incidence of prolonged ventilator time, and showed a trend towards lower mortality when compared to MS and conventional full sternotomy [25].

Additionally, we have noticed a statistically significant difference in cardiac index (CI), which was decreased during the MT procedure, probably due to reduced venous return from pulling the pericardium to visualize the aorta and the heart. The reduction of cardiac output during surgery had no impact on patient outcomes in our study.

In our study, maximal peak systolic velocity in middle cerebral artery blood flow velocity during cardiopulmonary bypass was an independent predictor of postoperative delirium. Ina recent study, Thudium at al [28]. clearly showed a critical role for cerebral perfusion in the pathogenesis of delirium following on-pump open-heart surgery, and recommended an individualized hemodynamic management, especially for at-risk populations. They defined cerebral hyperperfusion as the period when middle cerebral artery blood flow velocity during cardiopulmonary bypass was higher compared to the pre-bypass baseline value.

Postoperative delirium is independently associated with long-term mortality [29] and neurological decline [30]. Additionally, postoperative cognitive decline was previously correlated with MES number during cardiac interventions [31, 32]. In our study, we did not detect any relationship between intraoperative MES and neurological decline or postoperative delirium. Discrepancy between number of cerebral infarcts and clinical apparent stroke was previously shown in patients undergoing transcatheter aortic valve implantation, who were studied with cerebral MRI, both before and after (median 5 days) the procedure [33]. Overall, 24 out of 31 patients had a total of 131 new infarcts based on diffusion-weighted imaging. Only two patients (6%) were diagnosed with stroke on clinical grounds. Despite this data, overall health status and mental health were improved in these patients without apparent detriment from cerebral infarcts.

We found that age was associated with postoperative neurological decline, which is the most common adverse outcome following cardiac surgery [34]. These types of effects can be detected in different cognitive domains, including memory, attention and concentration, perception, motor performance, visuo-spatial perception, and language [35].

On the other hand, an observational study of 131 patients that included a healthy control group and two groups of patients with coronary artery disease (managed with either CABG or percutaneous coronary intervention) failed to show any significant differences in neurocognitive performance after 1 year of follow-up [36]. The study suggested that when CABG was performed using a standardized neuroprotective surgical technique (i.e., moderate hypothermia, epi-aortic scanning prior to placement of the aortic cannula, and maintenance of a mean arterial blood pressure greater than 60 mmHg during CPB), the choice of therapy did not have any impact on postoperative cognitive decline. Cognitive decline could be independent of surgical procedure and simply be a result of aging or another cardiovascular comorbidity [37]. The fact that two cohorts of elderly patients ended up with similar proportions of individuals at the same cognitive status at a certain time point did not necessarily imply a shared causal pathophysiologic mechanism [38].

We have found an independent relationship between postsurgical proinflammatory IL-6 levels and MES counts during surgery, but IL-6 levels were also independently associated with surgery length. In open-heart surgery, the contact of blood with artificial surfaces of the circuits of the CPB machine has been shown to cause perioperative SIRS [39]. In this manner, cellular (monocytes, neutrophils, lymphocyte, platelets, and endothelial cells) and humoral (fibrinolysis, intrinsic and extrinsic coagulation, and complement) inflammatory pathways are activated [39]. Inflammation is triggered by ischemia-reperfusion injury and the release of endotoxins. High levels of endothelial injury occur during an ischemic period, resulting in neutrophil activation and sequestration on reperfusion [4, 39]. Independent of leukocytes, production of toxic reactive oxygen species also occurs, leading to release of arachidonic acid metabolites, proinflammatory cytokines by ischemic cells (e.g., plasma tumor necrosis factor-alpha and interleukins like IL-1, IL-6, and IL-8), and activation of the humoral protein systems. After cerebral ischemic/reperfusion injury, microglial activation in the hippocampus results in release of multiple cytotoxic compounds, including reactive oxygen species (ROS), inflammatory cytokines and glutamate causing neuro-inflammation [40]. Further studies are necessary to determine whether neuroprotective and anti-inflammatory techniques will effect on neurocognitive decline in patients for minimal invasive AVR.

### Limitations

Our study had at least three major limitations. The first was that the study was observational, and patients were not randomized, so there were some age and weight differences between the MS and MT groups that could influence the results. However, previous studies have not shown any difference in age and weight between MS and conventional full sternotomy for AVR [24, 25, 27]. The second limitation was that our study was under powered and was not sufficient to definitively reject the hypothesis that there was no correlation between MES count and development of delirium and cognitive decline. The third major limitation was that the MS group included some patients with mechanical heart valves implanted while the MT group had only biological ones. To address this, we performed a sub analysis of MES load at different time periods only in patients receiving biological heart valves. There was no significant difference in MES load between MT and MS groups after exclusion of patients who received mechanical valves.

## Conclusions

To conclude with, our results indicate that MT represents a safe and efficient treatment strategy for surgical AVR yielding comparable results to MS approach in regards to intraoperative MES load. In our cohort of patients, MES load was namely associated only with CPB duration and not with surgical approach to the ascending aorta, clearly showing that less aggressive AVR performed through the second intercostal space can be safely performed even with limited exposure of the ascending aorta and the heart during the surgery. Since in our group of patients the postoperative neurologic decline was associated only with age, we were not able to confirm the relationship between postoperative neurological decline and intraoperative MES load.

## Supplementary Information

**Additional file 1 Table S1.** Microembolic signals, average maximal velocity in arteria cerebri media during surgery and values of serum IL 6. **Table S2.** Multiple linear regression model, showing predictors for total intraoperative count of microemboli as a dependent value. **Table S3.** Multiple linear regression with natural logarithm of IL 66 h after surgery as a dependent variable. **Table S4.** Binominal logistic regression with postoperative occurrence of delirium as a dependent variable. Estimates represent the log odds of “Delirium = 1” vs. “Delirium = 0”. **Table S5.** Multiple linear regression with ICU stay (days) as a dependent variable. **Table S6.** Result of postoperative cognitive function testing (number of points) as a depended variable in multiple linear regression model.

## Data Availability

The datasets used and/or analyzed during the current study are available on request.

## Abbreviations

ACE: Addenbrooke’s cognitive examination test
ANOVA: analysis of variance
AVA: aortic valve area
AVR: aortic valve replacement surgery
BSA: body surface area
ESC-EACTS: European Society of Cardiology and European Association for Cardio-Thoracic Surgery
CPB: cardiopulmonary bypass
GFR: glomerular filtration rate
CABG: coronary artery bypass graft
HITS: high intensity transient signals
ICU: intensive care unit
IL-6: interleukin 6
LVOT: left ventricular outflow tract
MAP: mean arterial pressure
MES: cerebral microembolic signals
mini - AVR: minimally invasive aortic valve replacement surgery
MS: mini-sternotomy
MT: mini-thoracotomy
NIRS: near-infrared spectroscopy
NYHA: New York Heart Association
TCD: Transcranial Doppler ultrasound
V max: peak systolic velocity
VTI: velocity time integral
