# Supplementary material for: Cerebral microemboli in mini-sternotomy compared to mini- thoracotomy for aortic valve replacement: a cross sectional cohort study
Source: J Cardiothorac Surg. 2021 May 24;16:142. doi: 10.1186/s13019-021-01509-8 (PMC8142614; doi:10.1186/s13019-021-01509-8)
Supplement: Supplementary file 1 — Additional file 1 Table S1. Microembolic signals, average maximal velocity in arteria cerebri media during surgery and values of serum IL 6. Table S2. Multiple linear regression model, showing predictors for total intraoperative count of microemboli as a dependent value. Table S3. Multiple linear regression with natural logarithm of IL 66 h after surgery as a dependent variable. Table S4. Binominal logistic regression with postoperative occurrence of delirium as a dependent variable. Estimates represent the log odds of “Delirium = 1” vs. “Delirium = 0”. Table S5. Multiple linear regression with ICU stay (days) as a dependent variable. Table S6. Result of postoperative cognitive function testing (number of points) as a depended variable in multiple linear regression model. [file 13019_2021_1509_MOESM1_ESM.docx]

**Additional Files**

**Additional table 1.** Microembolic signals, average maximal velocity in arteria cerebri media during surgery and values of serum IL 6

|  | **All (n=52)** | **MS (n= 25)** | **MT (n=27)** | **p-value*** |
| --- | --- | --- | --- | --- |
| MES-1^st^ period, n | 9.38±19.1 | 11.6±24.36 | 7.33±12.68 | 0.427 |
| MES-2^nd^ period, n | 3.65±8.11 | 1.08±2.18 | 6.04±10.60 | 0.026 |
| MES-3^rd^ period, n | 5.92±12.4 | 3.72±10.01 | 7.96±14.10 | 0.22 |
| MES-4^th^ period, n | 114±110 | 102.2±93.34 | 125.46±123.46 | 0.451 |
| MES-5^th^ period, n | 15.3±36.4 | 20.96±43.39 | 9.96±28.31 | 0.281 |
| MES-6^th^ period, n | 25±43.3 | 36.72±57.43 | 14.15±19.35 | 0.059 |
| Vmax 1^st^period, cm/s | 44.9±6.93 | 45.15±7.77 | 44.57±6.19 | 0.765 |
| Vmax 2^nd^ period, cm/s | 41.7±11.8 | 40.98 ±9.88 | 42.31±13.4 | 0.689 |
| Vmax 3^rd^ period, cm/s | 40.6±9.55 | 41.24±8.59 | 39.99±10.50 | 0.641 |
| Vmax 4^th^ period, cm/s | 45.8±4.98 | 45.06±3.13 | 46.43±6.22 | 0.328 |
| Vmax 5^th^ period, cm/s | 46±9.84 | 48.15±10.30 | 43.95±9.11 | 0.125 |
| Vmax 6^th^period,cm/s | 44.1±8.60 | 44.97±8.87 | 43.29±8.44 | 0.488 |
| IL 6 baseline,ng/L | 0.387±1.19 | 0.507±1.51 | 0.275±0.799 | 0.626 |
| IL 6 after 6 h, ng/L | 52±44.1 | 53.597±49.29 | 50.523±39.630 | 0.799 |
| IL 6 after 24 h,ng/L | 80.3±132 | 48.841±46.41 | 109.338±174.883 | 0.504 |

MT Mini thoracotomy, MS Mini sternotomy, MES- microembolic signals, V max-average maximal velocity in arteria cerebri media measured by TCD during surgery

Measurements time frame: ***1^st^ period***: from skin incision until aortic cannulation, ***2^nd^ period***: from aortic cannulation until start of CPB, ***3^rd^ period:*** the beginning of CPB until aorta clamping, ***4^th^period:***from beginning of aorta clamping until aorta clamp removed, ***5^th^ period:*** from aorta clamp removal until end of CPB, ***6^th^ period:*** end of CPB until end of surgery.

**Additional table 2.** Multiple linear regression model, showing predictors for total intraoperative count of microemboli as a dependent value

|  | | | | | | | | | | | | |
| --- | --- | --- | --- | --- | --- | --- | --- | --- | --- | --- | --- | --- |
|  | | | | | | **95% Confidence Interval** | | | |  | | |
| **Predictor** | | **Estimate** | | **SE** | | **Lower** | | **Upper** | | **P** | |  |
| Group: |  |  |  |  |  |  |  |  |  |  |  |  |
| 2 – 1 |  | -68.43 |  | 58.89 |  | -186.892 |  | 50.04 |  | 0.251 |  |  |
| CPB duration (min) |  | 5.64 |  | 2.47 |  | 0.677 |  | 10.60 |  | 0.027 |  |  |
| Surgeryduration (min) |  | -1.27 |  | 1.35 |  | -3.992 |  | 1.46 |  | 0.354 |  |  |
| Aorta clamping time |  | -5.72 |  | 3.41 |  | -12.582 |  | 1.13 |  | 0.100 |  |  |
|  | | | | | | | | | | | | |

MT Mini-thoracotomy (group 2), MS-Mini sternotomy (group 1), CPB cardiopulmonary bypass

**Additional table 3.** *Multiple linear regression with natural logarithm of IL 66 hours after surgery as a dependent variable*

|  | | | | | | | | | | | | | | | |  |  |
| --- | --- | --- | --- | --- | --- | --- | --- | --- | --- | --- | --- | --- | --- | --- | --- | --- | --- |
|  | | | | | | **95% Confidence Interval** | | | | |  | | | | |  |  |
| **Predictor** | | **Estimate** | | | **SE** | **Lower** | | | | **Upper** |  | | **P** | | |  |  |
| Surgery duration (min) |  | | -0.00232 |  | 0.00634 | |  | -0.01508 |  | 0.01045 |  |  | |  | 0.716 | |  |
| CPB duration (min) |  | | -0.00431 |  | 0.00814 | |  | -0.02070 |  | 0.01209 |  |  | |  | 0.599 | |  |
| Cell saver amount (L) |  | | 3.91e-4 |  | 3.85e-4 | |  | -3.85e-4 |  | 0.00117 |  |  | |  | 0.316 | |  |
| CRP 6h after surgery |  | | 0.01304 |  | 0.00240 | |  | 0.00821 |  | 0.01787 |  |  | |  | < .001 | |  |
| Total number of microemboli  during surgery |  | | 0.00141 |  | 6.65e-4 | |  | 6.61e-5 |  | 0.00275 |  |  | |  | 0.040 | |  |
|  | | | | | | | | | | | | | | | |  |  |

MT Mini thoracotomy , MS Mini sternotomy, CPB cardiopulmonary bypass, CRP C-reactive protein

**Additional table 4.** Binominal logistic regression with postoperative occurrence of delirium as a dependent variable. Estimates represent the log odds of “Delirium = 1” vs. “Delirium = 0”

|  | | 95% Confidence Interval | |  |
| --- | --- | --- | --- | --- |
| Predictor | Odds ratio | Lower | Upper | P value |
| Surgery duration (min) | 1.014 | 0.973 | 1.06 | 0.517 |
| V max avg | 1.383 | 1.031 | 1.83 | 0.03 |
| ACE-R after surgery | 0.906 | 0.820 | 1.00 | 0.053 |
| Intraoperative NIRS deviation | 1.022 | 0.901 | 1.16 | 0.741 |
| Group: |  |  |  |  |
| 2 – 1 | 1.174 | 0.155 | 8.91 | 0.876 |

MT Mini thoracotomy (group 2), MS Mini sternotomy (group 1), V max avg - average maximal velocity in arteria cerebri media measured by TCD during surgery, ACE-R Addenbrooke’s Cognitive Examination Test, NIRS near infrared spectroscopy

**Additional table 5:**Multiple linear regression with ICU stay (days) as a dependent variable

|  | | | | | | 95% Confidence Interval | | | |  | | |
| --- | --- | --- | --- | --- | --- | --- | --- | --- | --- | --- | --- | --- |
| Predictor | | Estimate | | SE | | Lower | | Upper | | P | |  |
| CPB duration (min) |  | -0.0383 |  | 0.01176 |  | -0.0620 |  | -0.0147 |  | 0.002 |  |  |
| Surgeryduration (min) |  | 0.0466 |  | 0.00927 |  | 0.0279 |  | 0.0652 |  | < .001 |  |  |
| Group: |  |  |  |  |  |  |  |  |  |  |  |  |
| 2 – 1 |  | -0.043 |  | 0.28596 |  | -0.6185 |  | 0.5320 |  | 0.880 |  |  |
| Positivebaseline IL 6 value |  |  |  |  |  |  |  |  |  |  |  |  |
| yes – no |  | 0.4650 |  | 0.32042 |  | -0.1796 |  | 1.1096 |  | 0.153 |  |  |

MT Mini thoracotomy (group 2), MS Mini sternotomy (group 1), CPB cardiopulmonary bypass

**Additional table 6**: Result of postoperative cognitive function testing (number of points) as a depended variable in multiple linear regression model

| 95% Confidence Interval | | | | | |
| --- | --- | --- | --- | --- | --- |
| Predictor | Estimate | SE | Lower | Upper | P |
| Surgeryduration (min) | -0.0240 | 0.0913 | -0.208 | 0.160 | 0.794 |
| CPB duration (min) | 0.0778 | 0.1175 | -0.159 | 0.314 | 0.511 |
| Age (years) | -0.3889 | 0.1312 | -0.653 | -0.125 | 0.005 |
| Group: |  |  |  |  |  |
| 2 – 1 | 3.4431 | 2.9928 | -2.578 | 9.464 | 0.256 |
